# *E-cadherin*基因启动子区甲基化与肺癌易感性的*meta*分析研究

**DOI:** 10.3779/j.issn.1009-3419.2013.07.04

**Published:** 2013-07-20

**Authors:** 园园 曾, 仍允 刘, 洪涛 张

**Affiliations:** 215123 苏州，苏州大学癌症分子遗传学实验室、苏州市癌症分子遗传学重点实验室 Soochow University Laboratory of Cancer Molecular Genetics, Medical College of Soochow University, Suzhou Key Laboratory for Molecular Cancer Genetic, Suzhou 215123, China

**Keywords:** E-cadherin, 甲基化, 肺肿瘤, 易感性, E-cadherin, Methylation, Lung neoplasms, Susceptibility

## Abstract

**背景与目的:**

上皮细胞钙粘蛋白E-cadherin在维持上皮的极性和结构完整性等方面起着重要作用，并与肿瘤的发生、浸润和转移密切相关。*E-cadherin*基因启动子区甲基化与肿瘤发生发展的关系一直是研究的热点，而该基因甲基化与肺癌易感性的关系尚存在争议。本研究旨在通过*meta*分析的方法更好地探讨*E-cadherin*基因启动子区甲基化与肺癌易感性之间的关系。

**方法:**

利用PubMed/MedLine和EMBASE数据库检索2013年3月以前的相关文献，由两位研究人员做独立文献筛选和资料提取，并交叉审核。使用优势比（odds ratio, OR）及95%置信区间（confidence interval, CI）衡量*E-cadherin*基因启动子区甲基化与肺癌易感性关系的强弱。

**结果:**

根据检索条件，共有13项研究（共涉及1, 288例实验样本）被纳入当前的*meta*分析。分析结果显示*E-cadherin*基因启动子区甲基化明显增加了肺癌发生风险（OR=4.04, 95%CI: 2.00-8.13, *P* < 0.001），且这种相关性同时存在于亚洲人群和高加索人群（OR=3.28, 95%CI: 1.20-8.92; OR=5.72, 95%CI: 2.40-13.62）。

**结论:**

*E-cadherin*基因启动子区甲基化与肺癌易感性之间存在明显相关性。

## Abstract (en)

**Background and objective:**

As a member of the cadherin superfamily, E-cadherin plays a critical role in the maintenance of cell polarity and integrity. And E-cadherin was reported to be down-regulated and closely related to tumor initiation, invasion and metastasis. There have been a number of studies investigating the methylation of *E-cadherin* promoter in relation to various cancers. However, the association between *E-cadherin* promoter methylation and lung cancer risk is controversial. The aim of the present study is to evaluate the association of *E-cadherin* promoter methylation with risk of lung cancer by conducting a systematic *meta*-analysis.

**Methods:**

Relevant studies were identified by searches of PubMed/MedLine and EMBASE databases before March 2013. Combined odds ratio (OR) and 95% confidence interval (CI) were used to assess the strength of the association between *E-cadherin* promoter methylation and lung cancer risk.

**Results:**

A total of 1, 288 samples from 13 independent studies were included in the *meta*-analysis. Overall, a significant association was observed between *E-cadherin* promoter methylation and lung cancer risk (OR=4.04, 95%CI: 2.00-8.13, *P* < 0.001). Subgroup analyses by ethnic revealed that lung cancer risk was increased for individuals carrying the methylated *E-cadherin* in Asian and Caucasian populations (OR=3.28, 95%CI: 1.20-8.92; OR=5.72, 95%CI: 2.40-13.62).

**Conclusion:**

The present *meta*-analysis indicates that the methylation of *E-cadherin* promoter is associated with risk of lung cancer.

肺癌是全世界范围内发病率最高的癌症类型，且是导致癌症患者死亡的首要因素^[1]^。尽管80%-90%的肺癌发生与吸烟相关，但肺癌的发生是一个多步骤、多因素参与的复杂的生物学过程，涉及多种遗传学和表观遗传学的改变^[2]^。作为一种重要的表观遗传修饰方式，DNA甲基化可以通过促进抑癌基因失活、原癌基因激活以及增加染色体不稳定性等多种方式，在肺癌等多种肿瘤发生过程中发挥关键作用^[3]^。抑癌基因启动子区CpG岛或启动子附近区域的异常甲基化引起转录抑制从而导致此类基因的活性降低甚至蛋白表达丢失是肺癌发生过程中的常见事件^[4]^。

*E-cadherin*基因（Cadherin-1, CDH1）位于染色体16q22.1，该基因编码的上皮细胞钙粘蛋白E-cadherin是一个分子量为120 kDa的跨膜糖蛋白，属于钙依赖性上皮细胞黏附分子，在维持上皮的极性和结构完整性等方面起着重要作用^[5]^。在肺癌等多种癌症患者体内E-cadherin表达降低，而抑制E-cadherin的表达下调可以阻止肿瘤细胞的转移，说明该基因与肿瘤的发生、发展和转移等密切相关，且在肿瘤发展过程中发挥着抑癌基因的作用^[6]^。研究^[7-9]^表明，在乳腺癌、膀胱癌和胃癌等多种肿瘤中，*E-cadherin*启动子区甲基化与E-cadherin表达下调或缺失有关，提示该基因甲基化参与了这些癌症的发生过程。然而，在肺癌中虽有多项研究曾涉及*E-cadherin*启动子区甲基化与肺癌发生的关系，但这些研究所得的结果并不完全一致。因此，为了更好地探讨*E-cadherin*启动子区甲基化与肺癌易感性之间的关系，我们收集、整理先前学者的所有研究数据，并进行*meta*分析。

## 1 材料与方法

### 1.1 数据收集

为了全面检索涉及*E-cadherin*基因启动子区甲基化与肺癌易感性关系的研究，我们使用“E-cadherin”、“CDH1”、“methylation”和“lung cancer”等作为关键词，利用PubMed/MedLine和EMBASE数据库检索发表于2013年3月以前的所有文献，并将综述类文献仔细阅读，以期从中得到相关的原始研究性文章的信息。

### 1.2 纳入标准及数据整理

如果某研究运用了病例-对照实验设计且*E-cadherin*基因启动子区甲基化频率可以从该文献中获取，那么该文章将被纳入当前的*meta*分析。如果两项或多项研究拥有重叠或重复的实验数据，那么我们只纳入包含更大样本量的研究。两位研究者（曾园园和刘仍允）独立检索数据库并阅读文献。在排除明显不符合纳入标准的文献后，阅读可能符合的文献全文，以确定其是否真正符合纳入标准。随后，两位研究者交叉核对纳入文献的结果，两人的纳入结果完全一致。对于符合纳入标准的文献，每一篇文献均按照作者、发表年份、实验人群所在的国家、实验人群的种族，以及患者和对照组人群的数量、甲基化检测方法以及实验材料的来源进行整理（表 1）。

**1:** 纳入*meta*分析的13项研究的基本特征 Characteristics of 13 studies included in the *meta*-analysis

Author	Year	Country	Ethnic	Cases	Controls	Methods	Materials
Zochbauer-Muller	2001	Australia	Caucasian	107	104	MSP	Tissue
Yanagawa	2003	Japan	Asian	75	75	MSP	Tissue
Russo	2005	US	Caucasian	49	27	MSP	Tissue
Tsou	2005	US	Caucasian	7	11	MSP	Tissue
Kim	2007	Korea	Asian	88	88	MSP	Tissue
Tan	2007	Singapore	Asian	20	10	MSP	Blood
Feng	2008	US	Mixed	49	49	MethyLight	Tissue
Wang	2008	China	Asian	95	95	MSP	Tissue
Wang	2008	China	Asian	28	12	3-D microarray	Tissue
Liu	2009	China	Asian	35	25	BSP	Tissue
Begum	2011	US	Mixed	76	30	qMSP	Tissue
Guzman	2012	Chile	Mixed	26	33	MSP	Tissue
Zheng	2012	China	Asian	37	37	MSP	Tissue

### 1.3 统计分析

*E-cadherin*基因启动子区甲基化与肺癌易感性关系的强弱程度用优势比（odds ratio, OR）及95%置信区间（confidence interval, CI）表示，而OR值是否有统计学意义采用*Z*检验进行分析。根据实验人群的种族、甲基化检测方法和实验材料来源的不同执行亚组分析。使用以卡方检验为基础的*Q*检验衡量样本的同质性^[10]^，并以*I*^2^值的大小反应多组研究之间同质性的程度（*I*^2^值越小，同质性越强）^[11]^。当各研究间具有较好同质性时，采用基于*Mantel-Haenszel*方法的固定效应模型来分析*meta*分析的结果; 反之，采用基于*DerSimonian and Laird*方法的随机效应模型进行分析^[12, 13]^。使用基于*Begg*检验为基础的漏斗图（Funnel plot）检测数据之间是否存在发表偏倚，采用*Egger*检验进行计算^[14]^。上述所有统计分析均采用STATA软件（Stata/SE version 10.1 for Windows; Stata Corp, College Station, Texas），*P* < 0.05认为差异具有统计学意义。

## 2 结果

### 2.1 被纳入的研究的基本信息

根据我们的检索条件及纳入标准，共有13项研究被纳入当前的*meta*分析^[15-27]^（表 1）。这13项研究共涉及1, 288例实验样本，包含692例肺癌样本和596例对照样本。其中7项研究（涉及378例肺癌样本和342例对照样本）源自亚洲人群，3项研究（163例肺癌样本和142例对照样本）源自高加索人群，另外3项研究（151例肺癌样本和112例对照样本）涉及两种以上的人群。此外，其中12项研究的研究对象为组织样本，另外1项为血液样本。在分析样本甲基化状态时，其中有9项研究采用了甲基化特异性PCR（MSP）的方法，另外4项使用了MethyLight分析、三维微列阵分析（3-D microarray）等其它实验方法。

### 2.2 *E-cadherin*基因启动子区甲基化与肺癌易感性

将收集到的13项研究的数据全部纳入*meta*分析，我们发现这些研究之间存在很强的异质性（*P* < 0.001，*I*^2^=74.9%，表 2），因此我们采用随机效应模型来分析*E-cadherin*基因启动子区甲基化与肺癌易感性的关系，结果发现*E-cadherin*基因甲基化可以明显增加肺癌的发病风险（OR=4.04，95%CI: 2.00-8.13，*P* < 0.001，图 1）。按照实验人群的种族进行亚组分析后，在亚洲人群和高加索人群中仍然可见*E-cadherin*基因甲基化与肺癌易感性呈明显正相关的现象（*P*=0.020，*P* < 0.001，表 2）。按照样本来源和甲基化检测方法进行亚组分析的结果表明：在组织来源的研究以及使用MSP为检测方法的研究中*E-cadherin*基因甲基化可以明显增加肺癌的发病风险。

**2:** *E-cadherin*基因甲基化与肺癌易感性 Summary of *E-cadherin* methylation and lung cancer risk

Variables	No.^a^	Cases/Controls	OR (95%CI)	*P*_*H*_^b^	*I*^2^ (%)
Total	13	692/596	4.04 (2.00-8.13)	< 0.001	74.9
Ethnicities					
Asian	7	378/342	3.28 (1.20-8.92)	< 0.001	77.9
Caucasion	3	163/142	5.72 (2.40-13.62)	0.056	65.4
Mixed	3	151/112	4.77 (0.96-23.70)	0.001	86.5
Testing materials					
Tissue	12	672/586	4.00 (1.94-8.24)	< 0.001	77.0
Blood	1	20/10	5.73 (0.28-117.65)	-	-
Testing methods					
MSP	9	504/480	4.40 (2.28-8.48)	0.023	54.9
Others	4	188/116	2.69 (0.41-17.69)	< 0.001	89.9
^a^No. of studies; ^b^*P* value of *Q* test for heterogeneity.					

**1:**
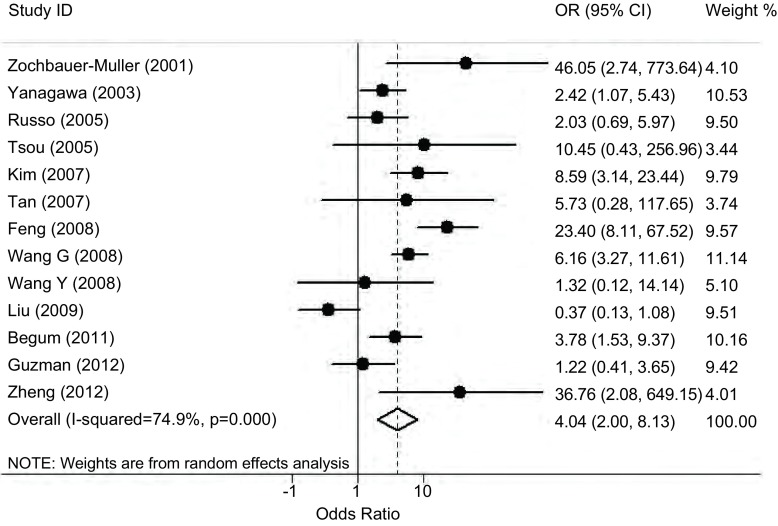
*E-cadherin*基因启动子区甲基化与肺癌易感性关系的*meta*分析 *Meta*-analysis of the association between *E-cadherin* promoter methylation and lung cancer risk

### 2.3 发表偏倚分析

使用OR值的自然对数及其标准误创建基于*Begg*检验为基础的漏斗图来衡量发表偏倚（图 2），该漏斗图呈现出良好的对称性，说明我们的*meta*分析并不存在发表偏倚。此外，*Egger*检验的结果证实了漏斗图的结果（*t*=0.39, *P*=0.707）。

**2:**
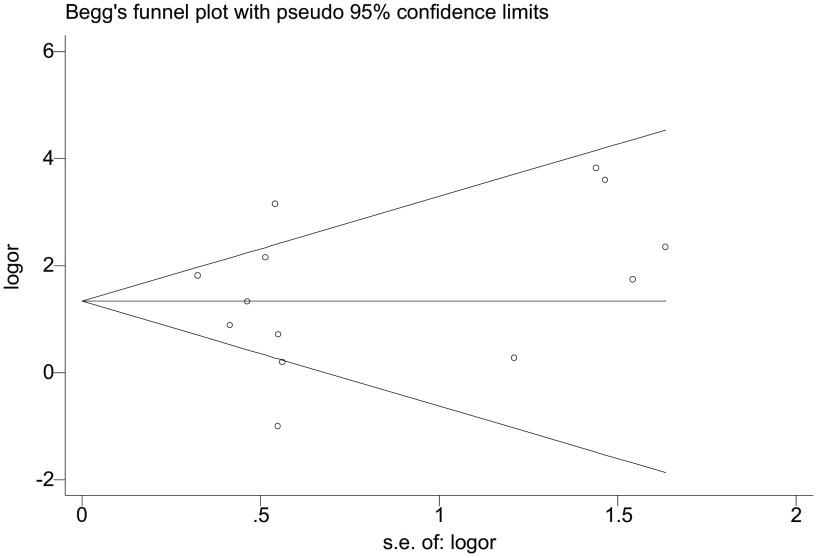
关于*E-cadherin*基因启动子区甲基化与肺癌发病风险研究的漏斗分析图 Funnel plot analysis on the association of *E-cadherin* promoter methylation with risk of lung cancer

## 3 讨论

抑癌基因启动子区异常甲基化导致抑癌基因转录失活的生物学过程参与了肺癌等多种癌症的发生。*E-cadherin*基因启动子区异常甲基化是导致E-cadherin表达下调或缺失的一个重要原因，并参与了乳腺癌、膀胱癌、子宫癌和胃癌等多种癌症的发生发展过程^[7-9, 28]^。有研究表明，在肺癌患者体内E-cadherin表达降低^[29]^。因此，*E-cadherin*基因甲基化可能是导致其蛋白表达降低的一个重要原因，进而改变了患肺癌的易感性，参与了肺癌发生。基于此，多位研究者探索了*E-cadherin*基因甲基化与肺癌发生的关系，然而这些研究得出的结论存在一定的分歧。

本研究采用*meta*分析的方法，首次系统性地分析了*E-cadherin*基因启动子区甲基化与肺癌易感性的关系。我们对先前发表的13项研究的数据（共包含692例肺癌样本和596例对照样本）进行分析，结果表明*E-cadherin*基因启动子区甲基化可以明显增加肺癌易感性，且我们的亚组分析数据表明这种相关性同时存在于亚洲人群和高加索人群。因此，我们推测*E-cadherin*与其它某些重要抑癌基因甲基化的作用模式相类似，即它们的启动子区甲基化参与肺癌发生过程在各类人种中具有普遍性。

除了根据人种进行亚组分析以外，我们还根据纳入研究的甲基化研究方法和研究材料进行了亚组分析。我们发现在使用MSP为检测方法的研究中*E-cadherin*基因启动子区甲基化增加了肺癌的发病风险，而使用其它检测方法的研究并不能表明*E-cadherin*基因甲基化与肺癌易感性相关，这可能是由于使用这些方法的研究所涉及的样本量较小而导致的。纳入当前*meta*分析的研究大多都采用组织样本为研究对象，只有1项研究（包含20例患者和10例对照者）选用了血液样本^[21]^，尽管这项研究不能够直接表明*E-cadherin*基因甲基化与肺癌易感性相关，我们不能排除使用大样本量血液样本的研究得到相关的可能性，因为在该研究中*E-cadherin*基因甲基化频率在肺癌患者和对照组中存在较大差异：分别为20%和0%。

检测所纳入的研究间的异质性是*meta*分析的重要组成部分，我们使用*Q*检验和*I*^2^值的大小对所涉及的研究是否具有异质性做了详细分析，在执行总体分析和亚组分析时根据异质性存在与否选择性使用随机效应或固定效应模型对*E-cadherin*基因甲基化与肺癌易感性的关系进行系统评价。除异质性分析以外，发表偏倚的检测也尤为重要。本研究中，我们运用基于*Begg*检验的漏斗图衡量发表偏倚情况，并使用*Egger*检验对漏斗图的结果进行验证，结果漏斗图和*Egger*检验显示出良好的一致性，这些均提示当前的*meta*分析不存在发表偏倚。

综上所述，当前的*meta*分析表明*E-cadherin*基因启动子区甲基化增加了肺癌发生风险。尽管我们的结论需要更大的样本量以及亚洲和高加索人群以外的样本中进行验证，但由于*E-cadherin*基因启动子区甲基化在肺癌发生过程中是一个频发事件，因此*E-cadherin*基因启动子区甲基化有望成为检测肺癌发生的一个潜在分子指标。
